# Effects of individualized positive end-expiratory pressure on intraoperative oxygenation in thoracic surgical patients: study protocol for a prospective randomized controlled trial

**DOI:** 10.1186/s13063-023-07883-z

**Published:** 2024-01-02

**Authors:** Xu-Ming Liu, Xin-Lu Chang, Jing-Yi Sun, Wen-Wen Hao, Li-Xin An

**Affiliations:** Department of Anesthesiology, Beijing Friendship Hospital, Capital Medical University, No. 95 Yongan Road, Xicheng District, Beijing, 100050 China

**Keywords:** Individualized positive end-expiratory pressure (iPEEP), One-lung ventilation (OLV), Intraoperative oxygenation, Postoperative pulmonary complications, Thoracic surgery

## Abstract

**Background:**

Intraoperative hypoxemia and postoperative pulmonary complications (PPCs) often occur in patients with one-lung ventilation (OLV), due to both pulmonary shunt and atelectasis. It has been demonstrated that individualized positive end-expiratory pressure (iPEEP) can effectively improve intraoperative oxygenation, increase lung compliance, and reduce driving pressure, thereby decreasing the risk of developing PPCs. However, its effect during OLV is still unknown. Therefore, we aim to investigate whether iPEEP ventilation during OLV is superior to 5 cmH_2_O PEEP in terms of intraoperative oxygenation and the occurrence of PPCs.

**Methods:**

This study is a prospective, randomized controlled, single-blind, single-center trial. A total of 112 patients undergoing thoracoscopic pneumonectomy surgery and OLV will be enrolled in the study. They will be randomized into two groups: the static lung compliance guided iPEEP titration group (Cst-iPEEP Group) and the constant 5 cmH_2_O PEEP group (PEEP 5 Group). The primary outcome will be the oxygenation index at 30 min after OLV and titration. Secondary outcomes are oxygenation index at other operative time points, PPCs, postoperative adverse events, ventilator parameters, vital signs, pH value, inflammatory factors, and economic indicators.

**Discussion:**

This trial explores the effect of iPEEP on intraoperative oxygenation during OLV and PPCs. It provides some clinical references for optimizing the lung protective ventilation strategy of OLV, improving patient prognosis, and accelerating postoperative rehabilitation.

**Trial registration:**

www.Chictr.org.cnChiCTR2300073411. Registered on 10 July 2023.

## Background

Thoracoscopic surgery requires one-lung ventilation (OLV), which involves isolation of the healthy lung and atrophy of the surgical lung, to better expose the operative field and minimize intraoperative risks to patients. OLV, on the other hand, is prone to ventilator-induced lung injuries, such as volutrauma, atelectrauma, and oxygen toxicity [1]. Surgical injury and OLV are also associated with severe inflammatory cytokine release due to the abundant immune cells in the pulmonary endothelium and alveoli [2]. In response to pro-inflammatory cytokines, excessive accumulation of neutrophils leads to increased pulmonary vascular permeability. These reactions may further exacerbate lung injury during OLV, resulting in persistent intraoperative hypoxia and a significant increase in postoperative pulmonary complications (PPCs), including pulmonary atelectasis, acute lung injury, acute respiratory distress syndrome, etc. Some studies have shown that the incidence of PPCs in patients undergoing pulmonary resection surgery can be as high as 10% to 50% [3, 4], which seriously affects the prognosis and slows the recovery of patients [5]. Therefore, it is strongly recommended to perform lung-protective ventilation during thoracic surgery.

Lung protective ventilation strategy (LPVS) including low tidal volumes (V_T_), positive end-expiratory pressure (PEEP), and alveolar recruitment maneuvers (ARM) [6] has been demonstrated to may reduce the ventilation/perfusion ratio imbalance, improve intraoperative oxygenation during mechanical ventilation, and reduce the occurrence of PPCs [7]. And individualized PEEP (iPEEP), which is the most recommended LPVS in the 2019 international expert panel-based consensus, has the advantage of better increasing static lung compliance, improving oxygenation, and reducing ventilator-associated lung injury and PPCs, as compared to constant PEEP [8]. However, few studies have shown the effectiveness of iPEEP in thoracoscopic surgery, and its performance during OLV remains unclear.

In recent years, studies on perioperative LPVS in thoracic surgery patients have always focused on the effects of lower V_T_ or different levels of PEEP on PPCs. However, the results of these studies are controversial. A multicenter retrospective observational analysis of patients receiving OLV showed there is no independent association between low V_T_ and PPCs [9]. Spadaro S et al. employed different levels of PEEP (0, 5, and 10 cmH_2_O) in patients on OLV in a randomized controlled trial and found that high levels of PEEP improved pulmonary function [10]. However, this relatively fixed high PEEP is not appropriate for all patients [7]. Some alveoli will lose their ventilatory function due to over-expansion during mechanical ventilation, which will also reduce the efficiency of ventilation and impair pulmonary function. Park M et al. found that the application of driving pressure-guided ventilation during OLV reduced the incidence of PPCs compared with conventional lung-protective ventilation [11]. However, although driving pressure-guided PEEP is easy to apply intraoperatively, it is susceptible to many factors such as variations in body position. The potential causes of elevated driving pressure cannot be easily assessed intraoperatively to maintain low driving pressure in time. Electrical impedance tomography (EIT) guided iPEEP titration can monitor the ventilation and perfusion of the local lung in real time [12, 13], but it is expensive, complicated to operate, and difficult to provide intraoperative bedside guidance. So it is not conducive to the popularization of iPEEP.

Using the static pulmonary compliance (Cstat) guided titration method, the optimal equilibrium between lung hyperinflation and atelectasis can be achieved as much as possible [14]. We applied this method to obese patients previously and found that titrated iPEEP guided by optimal Cstat not only did not increase driving pressure but also significantly reduced the incidence of postoperative atelectasis in obese patients undergoing laparoscopic surgery compared with constant PEEP [15, 16]. Battaglini D et al. [17] also suggested that the ideal approach to titrate PEEP should be based on the optimal Cstat.

Therefore, we will conduct a prospective randomized controlled trial in thoracic surgery to compare the clinical outcomes such as intraoperative oxygenation and the incidence of PPC, during OLV with conventional lung-protective ventilation using fixed PEEP versus Cstat-guided iPEEP. We hypothesized that Cstat-guided iPEEP ventilation would improve intraoperative oxygenation during OLV and reduce PPCs in thoracic surgery compared with fixed PEEP ventilation.

## Methods/design

### Objectives and design

**Fig. 1:**
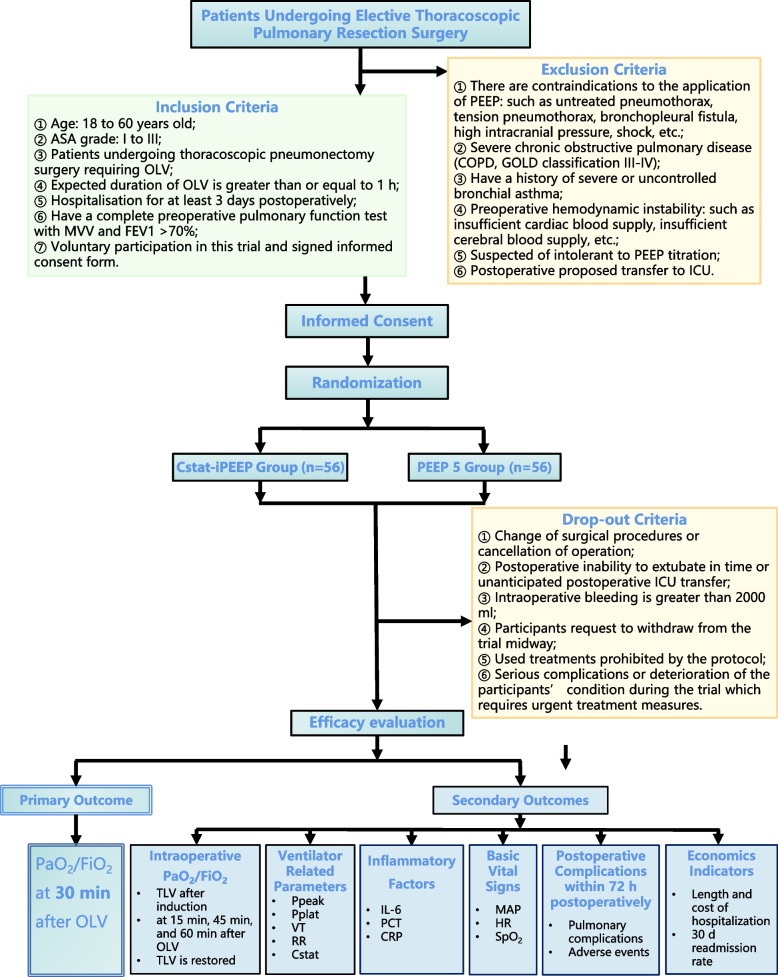
Flowchart

This study is a prospective, randomized, controlled, single-blind trial, that aims to test the hypothesis that Cstat-guided iPEEP ventilation during OLV may improve intraoperative oxygenation and decrease the incidence of PPCs in thoracic surgery patients. 112 patients will be randomly divided into two groups at a ratio of 1:1: the Cstat-guided iPEEP titration group (Cstat-iPEEP Group, *n*=56) or the constant 5 cmH_2_O PEEP group (PEEP 5 Group, *n*=56). (see Consolidated Standards of Reporting Trials [CONSORT] diagram, Fig. 1). Patient recruitment will end on December 31, 2024.

This study will be conducted at the Department of Anaesthesiology, Beijing Friendship Hospital, Capital Medical University. It has been approved by the Ethics Committee of the Beijing Friendship Hospital Affiliated to Capital Medical University (the approval number is 2023-P2-118-02) and has been registered at the Chinese Clinical Trial Registry (the registration number is ChiCTR2300073411). Under hospital regulations, compensation and post-trial care will be provided to those injured as a result of their participation in the experiment.

### Blinding and randomization

This trial is a study in which patients and evaluators are blinded, while implementers are not blinded. A statistician will generate the allocation sequence using a computer-generated table of numbers and an assistant (always a graduate student) prepare corresponding hidden envelopes based on the grouping sequence. The chief anesthetist (A) will screen patients and enroll them based on inclusion/exclusion criteria. He will explain the process, benefits, and risks of the trial to patients, as well as the collection of participant data. In order to encourage patients to participate in this study, we will inform them of additional benefits, such as free blood gas analysis. After the patients agree, they will sign an informed consent form with the anesthetist. The anesthetist (A) will obtain the covered grouping envelope from the assistant and open it to obtain grouping information. Patients, surgeons, and data collection observers will be unaware of the group assignments.

### Study population

Patients scheduled for thoracoscopic pulmonary resection surgery will be screened and recruited during routine preoperative assessment. Participants who meet the following inclusion criteria will be eligible: ① Age: 18 to 65 years old; ② ASA grade: I to III; ③ Patients undergoing thoracoscopic pneumonectomy surgery requiring OLV; ④ Expected duration of OLV is greater than or equal to 1 h; ⑤ Hospitalization for at least 3 days postoperatively; ⑥ Have a complete preoperative pulmonary function test with MVV and FEV_1_ >70%; ⑦ Voluntary participation in this trial and signed informed consent form. In addition to sufficient communication, some small gifts will also be provided to patients as rewards to facilitate postoperative follow-up cooperation.

Exclusion criteria are as follows: ① There are contraindications to the application of PEEP: such as untreated pneumothorax, tension pneumothorax, bronchopleural fistula, high intracranial pressure, and shock; ② Severe chronic obstructive pulmonary disease (COPD, GOLD classification III–IV); ③ Have a history of severe or uncontrolled bronchial asthma; ④ Preoperative hemodynamic instability: such as insufficient cardiac blood supply, and insufficient cerebral blood supply; ⑤ Suspected of intolerant to PEEP titration; ⑥ Postoperative proposed transfer to ICU.

Participants who were included in this trial may also drop out because of the following criteria: ① Change of surgical procedures or cancelation of operation; ② Postoperative inability to extubate in time or unanticipated postoperative ICU transfer; ③ Intraoperative bleeding is greater than 2000 ml; ④ Participants request to withdraw from the trial midway; ⑤ Used treatments prohibited by the protocol; ⑥ Serious complications or deterioration of the participants’ condition during the trial which requires urgent treatment measures.

### Standard anesthesia procedure

To avoid interference with the experimental results, the anesthetist and the surgical team were relatively fixed and anesthetized according to the clinical routine. The following strategy is recommended (Fig. 1):

1. According to the clinical routine, patients will be monitored for the following parameters upon entering the operating room: invasive blood pressure, electrocardiogram, bispectral index, pulse oximetry, and urine output. Patients without poorly controlled diabetes mellitus (fasting blood glucose >11.0 mmol/L), and peptic ulcer, were routinely given 40 mg of methylprednisolone sodium succinate before anesthesia induction.
2. Rapid anesthesia induction was performed by applying midazolam (0.05 mg/kg), etomidate (0.3 mg/kg), sufentanil (0.5 μg/kg), and cisatracurium (0.2 mg/kg). After induction, we used a visual laryngoscope to intubate a left double-lumen bronchial catheter (35F for females and 37F for males), and the final intubation position was identified by fibreoptic bronchoscopy. The thoracic paravertebral blockade was conducted under ultrasound guidance as part of the postoperative analgesic plan.
3. Intraoperative anesthesia was maintained with intravenous injecting of isoproterenol (4–6 mg/(kg h)), and remifentanil (0.1–0.2 μg/(kg min)).
4. Intraoperative hemodynamics is managed based on surgical procedures and blood loss.
5. The paravertebral nerve block was routinely administered preoperatively for postoperative analgesia to ensure a visual analog scale pain score < 3.
6. Postoperative physiotherapy will be performed, including encouraging patients to get out of bed as early as possible, assisting patients with coughing and expectoration, and urging patients to practice deep breathing.

Anesthesia-related data should be collected and analyzed thoroughly. Anesthesia care and associated treatment must adhere to clinical routines. Catheterize the patient before surgery.

### Mechanical ventilation

The ventilator settings are as follows: The ventilator was used in PCV-VG mode, with a set plateau pressure (Pplat) of 30 cmH_2_O, the fraction of inspiration oxygen (FiO_2_) of 50%, a tidal volume of 6–7 ml/PBW (predicted body weight, 6 ml/PBW for OLV and 7 ml/PBW for TLV), a respiratory rate of 12–15 breaths/min (to maintain a P_ET_CO_2_ of 35–45 cmH_2_O), and an inspiratory to expiratory ratio (I: E) of 1:2.

### Intervention

**Fig. 2:**
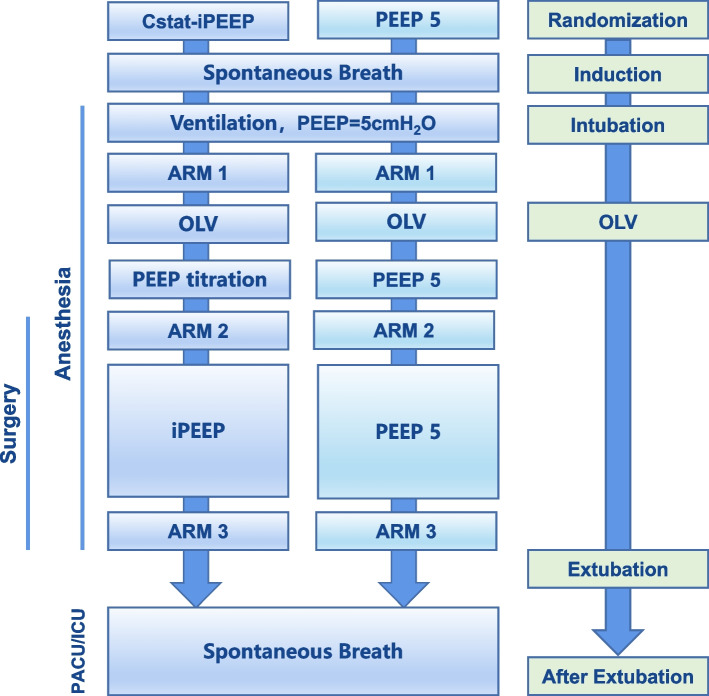
Trial procedures

After tracheal intubation and mechanical ventilation, PEEP was maintained at 5 cmH_2_O for 5 min, and baseline respiratory parameters were recorded. All patients (both groups) received ventilator-driven alveolar recruitment maneuvers (ARM) 5 min after tracheal intubation [11], and the same procedure was repeated after the completion of PEEP titration during OLV and before extubation (Fig. 2).

#### ARM procedure

**Fig. 3:**
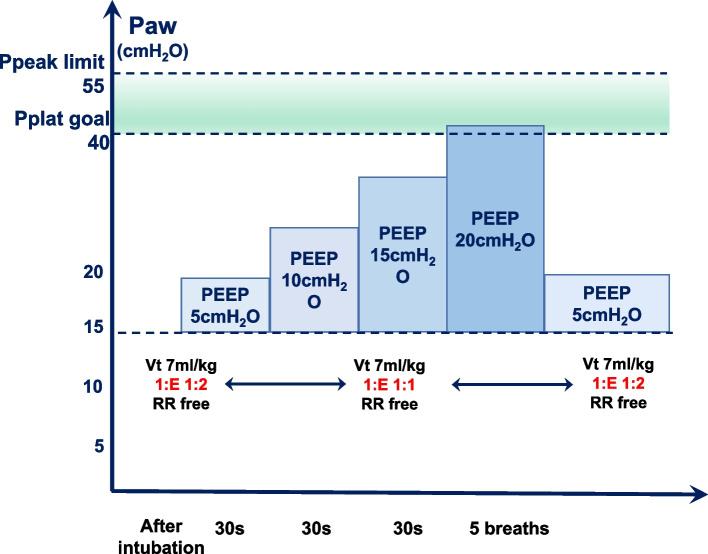
Flowchart of ARM

ARM is performed in the following steps (Fig. 3):

1. In the pressure-controlled ventilation (PCV) mode of the ventilator, the driving pressure is restrained at 15–20 cmH_2_O, and the peak airway pressure (Ppeak) is 55 cmH_2_O.
2. PEEP was gradually raised from 5 cmH_2_O to 20 cmH_2_O, increasing by 5 cmH_2_O each time for 30–60 s. The ARM procedure will be terminated if Pplat reaches 40 cmH_2_O.
3. During the ARM, V_T_ is set to 7ml/kg and I: E to 1:1.
4. During the ARM, all patients received a standardized fluid regimen and vasopressor medications to attenuate short-term hemodynamic depression caused by ARM and to maintain mean arterial pressure (MAP) ≥ 65 mmHg. When there is a downward trend in MAP, accelerate the input of crystalloid 100 ml. If the decline in MAP is more than 20% of the base value, immediately push methotrexate 1 mg intravenously, then observe the recovery of MAP, and repeat the measure if necessary. If the decline in MAP lasts for longer than 5 min, norepinephrine will be continuously injected intravenously. After completing all the measures, we recorded all operating steps and drug dosage.

#### IPEEP titration

After the first ARM is performed, OLV will be started after the lateral position. The Cstat-iPEEP Group will be titrated to iPEEP based on optimal Cstat. And for the PEEP 5 Group, a constant 5 cmH_2_O PEEP will be set and maintained until the end of the surgery.

**Fig. 4:**
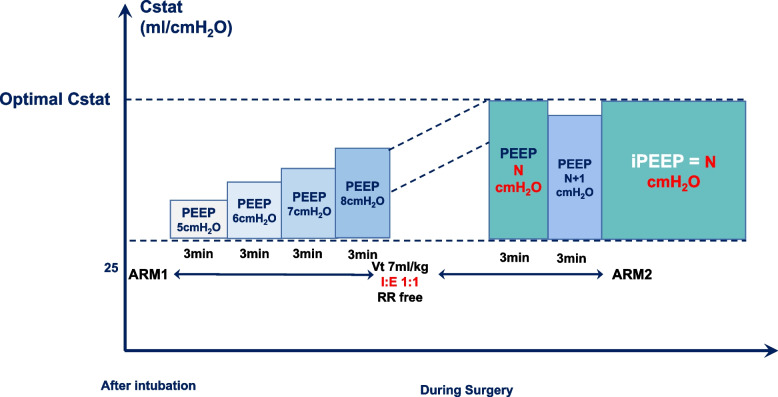
Flowchart of Cstat-guided PEEP titration

In the Cstat-iPEEP Group, the patient's iPEEP is set through the following procedure (Fig. 4):

1. The first ARM (ARM1) is performed 5 min after intubation.
2. V_T_ is set to 7ml/PBW, I: E to 1:1, respiratory rate to 12–15 breaths/min.
3. Change V_T_ to 6 ml/PBW for OLV.
4. Immediately after establishing OLV, titration of PEEP was initiated. The initial PEEP was set to 5 cmH_2_O, then the PEEP was increased at steps of 1 cmH_2_O, and each PEEP level was sustained for 3 min. While adjusting the PEEP, Cstat was calculated using the formula (Cstat = V_T_/(Pplat-PEEP)) until the calculated Cstat showed a declining trend. The PEEP corresponding to the calculated maximum Cstat was set as the optimal iPEEP for this patient.
5. The upper limit of PEEP is set to 20 cmH_2_O.
6. Thirty minutes after obtaining the iPEEP, we performed the second ARM (ARM2).
7. The third ARM (ARM3) is performed before extubation.

### Study endpoints

The primary outcome in this trial was the oxygenation index (OI = PaO_2_/FiO_2_) at 30 min after OLV and PEEP titration. We will draw 0.5 ml of the patient’s arterial blood at this time point for blood gas analysis (ABG) to obtain the OI.

The secondary outcomes include the oxygenation index at other intraoperative time points, PPCs, postoperative adverse events, vital signs, ventilator-related parameters, pH value, inflammatory factors, and economic indicators.

1. OI at other intraoperative time points: We will draw 0.5 ml of arterial blood for ABG at 15 min, 45 min, and 60 min after OLV and PEEP titration, respectively, and at the time of resumption of TLV, and calculate the OI at this time as secondary outcomes.
2. Postoperative pulmonary complications (PPCs): We will record pulmonary complications occurring within 72 h postoperatively and the occurrence of one of the following will be defined as positive PPCs [5].
  1. Pulmonary infection: The clinical pulmonary infection score (CPIS) will be used to assess pulmonary infection, and a CPIS score greater than or equal to 6 is defined as the presence of a postoperative lung infection in the patient [18]. We will record the temperature, leukocyte count, tracheal secretions, oxygenation index (OI), chest X-ray, and tracheal secretion culture of patients in the preoperative, 24 h postoperative, 48 h postoperative, and 72 h postoperative periods to calculate the score (Table 1);
  2. ARDS: Based on the Berlin definition, we designate OI ≤ 300 mmHg as ARDS;
  3. Respiratory failure: PaO_2_ < 60 mmHg or SpO_2_ < 90%;
  4. Pulmonary embolism: Diagnosis confirmed by CT or pulmonary arteriography;
  5. Pleural effusion or pneumothorax confirmed by imaging;
  6. Atelectasis: Confirmed by X-ray or CT;
  7. Aspiration pneumonia: Confirmed by bronchoscopy;
  8. Tracheospasm or bronchospasm: An experienced anesthetist or respiratory physician will determine whether the patient has developed tracheospasm or bronchospasm based on the patient's history, symptoms, and signs;
  9. Unanticipated respiratory support: The patient requires non-invasive or invasive mechanical ventilation.
3. Postoperative adverse events: one of the following occurring within 72 h after surgery was considered an adverse event.
  1. Increase or decrease in blood pressure: the fluctuation of more than 20% of the preoperative blood pressure;
  2. Tachycardia or bradycardia: the fluctuation of more than 20% of the preoperative heart rate;
  3. Newly detected arrhythmia;
  4. Postoperative bleeding: bleeding volume > 500 ml and < 2000 ml;
  5. Postoperative fever: temperature > 38 °C;
  6. Transient ischemic attack (TIA) or stroke.
4. Vital signs, ventilator-related parameters, and the pH value: vital signs, ventilator-related parameters, and the pH value were recorded at two-lung ventilation (TLV) after anesthesia, after 15 min of OLV, after 30 min of OLV, after 45 min of OLV, after 60 min of OLV, and at the resumption of TLV. Vital signs included mean arterial pressure, heart rate, and SpO_2_. Ventilator-related parameters included peak airway pressure, Pplat, tidal volume, respiratory rate, and static pulmonary compliance calculated using the formula. The pH value can be measured along with the OI during ABG.
5. Inflammatory factors: venous blood was drawn from the patients preoperatively, 24 h postoperatively, and 72 h postoperatively to test interleukin 6 (IL-6), C-reactive protein (CRP), and procalcitonin (PCT).
6. Economic indicators: economic indicators include the length and cost of hospitalization, and readmission rates within 30 days.

**Table 1:** Clinical pulmonary infection score (CPIS) [18]

Parameter		Points
Temperature(°C)	36-38	0
	38-39	1
	> 39 or < 36	2
Leukocyte count (×10^9^/L)	4-10.99	0
	11-17	1
	> 17 or < 4	2
Tracheal secretions (Shape and volume of secretions in 24 h)	None or a few	0
	Moderate to large, and non-purulent	1
	Moderate to large, and purulent	2
Oxygenation index (mmHg)	> 240 or ARDS	0
	≤240 and no ARDS	2
Chest X-ray	No infiltrate	0
	Diffuse or patchy infiltrate	1
	Localized infiltrate	2
Tracheal secretion culture	No growth	0
	Pathogenic growth	1
	The same pathogen was cultured twice, or the Gram stain was consistent with the culture results	2

### Adverse effects

If uncontrollable adverse events occur during or after surgery, such as an unstable circulatory system during surgery, persistent high airway pressure (Ppeak>40cmH_2_O), excessive blood loss during surgery, difficulty in extubation, and return to ICU after surgery, we will terminate the trial. And the incident will be recorded by the anesthetist responsible for the intervention and reported to the relevant department personnel within 48 h in accordance with hospital administrative rules.

Because the lung protection procedures used in this trial are all within the scope of clinical routine operations, there is no anticipated harm and compensation for trial participation. If any harm to a patient occurs that is unexpected and may be caused by trial interference factors, the trial will be terminated and reported promptly after the hospital expert committee and SRMC determine that it is accurately related to the trial procedure, and compensation will be provided according to regulations.

### Data collection and monitoring

**Fig. 5:**
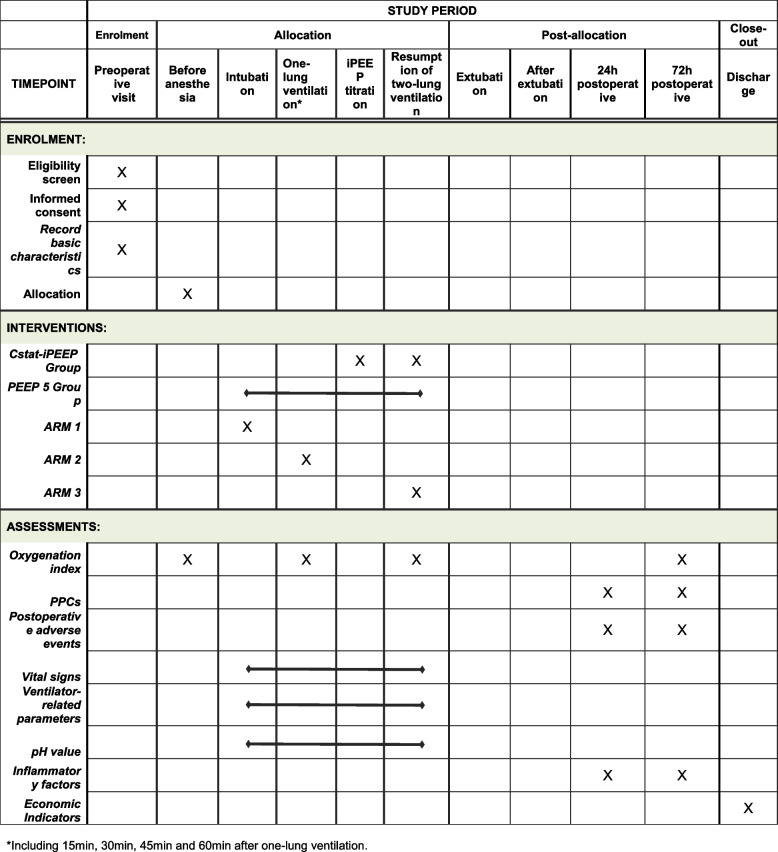
Standard protocol items study schedule

The anesthetist who performed the PEEP titration recorded the patient's basic characteristics as well as any relevant data throughout the surgery and after transfer to the post-anesthesia care unit (PACU). A physician collects data after the patient is transferred to the ward (Fig. 5). All raw data will be fully documented on the case report form (CRF). Cases with incomplete data are also recorded. The chief anesthetist will review trial conduct on a weekly basis. The CRF will be available on the clinical trial registration website of this trial (https://www.chictr.org.cn/showproj.html?proj=195633).

The scientific research management committee (SRMC) consists of an anesthetist, a statistician, and a scientific researcher. The functions of SRMC are data analysis and management, results adjudication, and protocol improvement. The anesthetist in SRMC will check the implementation of the project every month. The original data and results will be submitted to SRMC every 3 months, and electronic information will be entered into a password-protected email that will not be shared with the public until the results are published. The SRMC is independent from the sponsor and competing interests. The ethics committee will conduct inspections every year and at the end of the trial. If we update the protocol, we will submit the amendment to the ethics committee and clinical trial registry, and inform the sponsors, etc. All original records (informed consent forms, CRF, and related documents) will be stored and retained for 10 years to facilitate the retention and complete follow-up of participants, and then destroyed according to hospital standards.

### Sample size calculation

The primary outcome is the oxygenation index (OI) at 30 min after PEEP titration. From our pre-trial, we calculated the OI as 250.1±107.0 (*n*=10) for the Cstat-iPEEP Group and 189.9±74.5 (*n*=11) for the PEEP 5 Group. By performing the Student’s *T*-test with PASS 15.0 software, we estimated the minimal sample size of 102 patients with a power of 90% at a two-sided significance level of *P*=0.05. Adding a 10% drop-out rate, we would recruit a total of 112 cases, 56 in each group.

### Statistical analysis

SPSS 26.0 will be used to analyze the data. All quantitative indicators that conform to normal distribution are described by mean ± standard deviation, and those that do not conform are described by median (quartiles). Continuous variables, such as oxygenation index, anesthetic doses, procedure time, awake time, etc., will be analyzed using the Student’s *T*-test or Mann-Whitney *U*-test. A repeated analysis of measurement variance will be used to analyze data from repeated measures such as systolic blood pressure, heart rate, etc. Qualitative indicators, such as the incidence of PPCs, will be described in terms of frequencies (percentages) and analyzed using the chi-square test or Fisher’s test. Logistic regression analysis will be applied to identify risk factors for the occurrence of PPCs. We will conduct subgroup analysis for different surgical sides to clarify the different protective effects of iPEEP on left or right lung resection surgery. In the subgroup analysis, the oxygenation index will be analyzed using the Student’s *T*-test or Mann-Whitney *U*-test.

To avoid data loss due to time constraints, such as changes in circulation during iPEEP titration, we will use a mobile phone to record the entire process during surgery to supplement the incomplete recorded data. If there is still missing data, the average interpolation method will be used. Once the study is dropout, these cases will be reported. Intention-to-treat (ITT) includes all populations receiving treatment, including those with missing data obtained through mean imputation. Pre-protocol refers to the population with complete data records and no missing data. Both ITT and pre-protocol will be analyzed. For ITT analysis, data from all patients in the randomization group will be processed. If a large portion of patients have not received randomized intervention or lost follow-up, analysis will be conducted according to the pre-protocol to evaluate the primary outcome.

When half of the cases are included, the SRMC statistician will unblind and conduct an interim analysis. The main anesthetist in charge of this trial will share the interim results.

## Discussion

This is a prospective, single-blind, randomized, controlled trial. It aims to confirm whether Cstat-guided iPEEP ventilation would improve intraoperative oxygenation during OLV and reduce PPCs after thoracic surgery compared with fixed PEEP ventilation.

Decreased intraoperative oxygenation, which can be caused by a variety of factors, adversely affects patients both intraoperatively and postoperatively. During OLV, the lung on the ventilated side can experience alveolar hyperexpansion during mechanical ventilation, causing a massive release of inflammatory factors, generating an inflammatory response, and destroying the heterogeneity between lung units. Thus, after the restoration of TLV, the ability of the alveoli to regulate pressure decreases, increasing poorly ventilated lung tissue and causing local atelectasis [19]. On the other hand, the normal relationship between functional residual capacity and closed volume is disrupted in the surgical lung due to surgical operations such as pulmonary atrophy and surgical pneumothorax, causing physiological changes such as increased alveolar-arterial oxygen partial pressure difference and hypoxic pulmonary vasoconstriction (HPV), which result in an intraoperative decrease in oxygenation and imbalance of ventilation/perfusion ratio [1]. The incidence of SpO_2_ of less than 90% during OLV can be as high as 5% [20].

Therefore, choosing an appropriate ventilation strategy is crucial for patients undergoing OLV, and appropriate LPVS is important for improving the prognosis of patients, enhancing their postoperative quality of life, and better practicing the concept of enhanced recovery after thoracic surgery (ERATS).

LPVS includes low V_T_, ARM, PEEP, permissive hypercapnia, and low FiO_2_. A secondary analysis of related studies by Simon P et al. [21] confirms that PEEP leads to a more even distribution of gas in the lungs, further improving intraoperative oxygenation. Some studies have also pointed out that PEEP in TLV requires at least 10 cmH_2_O to reduce the incidence of PPCs such as atelectasis [22, 23], but high levels of PEEP are prone to cause hemodynamic disturbances such as increased intrathoracic pressure and reduced cardiac output.

Therefore, the concept of individualized PEEP was born, which means that PEEP is set up individually according to different individuals, different diseases, and different disease courses to achieve better lung protection effects. Many available studies have confirmed that iPEEP is effective in enhancing intraoperative oxygenation, improving lung compliance, and reducing PPCs during TLV [24–27].

At the end of OLV, re-expansion of the atrophied side of the lung induces local ischemia-reperfusion injury with the release of more inflammatory factors and oxygen-free radicals [28]. Misthos P et al. suggested that the longer the OLV lasts, the more oxygen-free radicals are released, and the greater the probability of causing lung injury and pulmonary edema [29, 30]. In this regard, we will collect data on inflammatory factors in patients at 24 h and 72 h postoperatively to explore possible ways of applying lung protection with iPEEP.

In conclusion, we believe that this trial will verify the hypothesis that Cstat-guided iPEEP ventilation will improve intraoperative oxygenation and decrease PPCs. It will provide some clinical evidence for optimizing the LPVS of OLV, improving patient prognosis, and accelerating postoperative rehabilitation.

## Trial status

Our study protocol was refined on 2023.08.25 with version number 4.2/2023.08.25. The first version was developed on April 23, 2023. Recruitment started on August 28, 2023, and the first participant was successfully recruited on August 29, 2023. The recruitment and trial are expected to end in December 2024. The recruitment will end on December 31, 2024. To date, 2 participants have been recruited. This trial is still going on.

## Data Availability

Upon completion of the study and publication of the results, the data and trial results will be made available to the public (including participants) by contacting the corresponding author via email.

## Abbreviations

OLV: One-lung ventilation
PPCs: Postoperative pulmonary complications
LPVS: Lung protective ventilation strategy
V_T_: Tidal volumes
PEEP: Positive end-expiratory pressure
ARM: Alveolar recruitment maneuvers
iPEEP: Individualized positive end-expiratory pressure
EIT: Electrical impedance tomography
Cstat: Static pulmonary compliance
CONSORT: Consolidated Standards of Reporting Trials
ASA: American Society of Anesthesiologists
MVV: Maximal voluntary ventilation
FEV_1_: Forced expiratory volume in the first second
COPD: Chronic obstructive pulmonary disease
GOLD: Global Initiative for Chronic Obstructive Lung Disease
ICU: Intensive Care Unit
PCV-VG: Pressure-controlled ventilation-volume guaranteed
Pplat: Plateau pressure
FiO_2_: The fraction of inspiration oxygen
PBW: Predicted body weight
P_ET_CO_2_: Pressure of end-tidal CO_2_
I: E: Inspiratory to expiratory ratio
PCV: Pressure-controlled ventilation
Ppeak: Peak airway pressure
MAP: Mean arterial pressure
OI: Oxygenation index
ITT: Intention-to-treat
PaO_2_: Pulmonary arterial oxygen tension
ABG: Blood gas analysis
pH: Potential of hydrogen
CPIS: Clinical pulmonary infection score
ARDS: Acute respiratory distress syndrome
SpO_2_: Percutaneous arterial oxygen saturation
CT: Computed tomography
TIA: Transient ischemic attack
TLV: Two-lung ventilation
IL-6: Interleukin 6
CRP: C-reactive protein
PCT: Procalcitonin
PACU: Post-anesthesia care unit
CRF: Case report form
SRMC: Scientific research management committee
HPV: Hypoxic pulmonary vasoconstriction
ERATS: Enhanced recovery after thoracic surgery
